# Building Strong Primary Healthcare Systems in Greece

**DOI:** 10.7759/cureus.41333

**Published:** 2023-07-03

**Authors:** Maria Kampouraki, Panagiotis Paraskevopoulos, Evelin Fachouri, Sotiris Terzoudis, Dimitrios Karanasios

**Affiliations:** 1 Family Medicine, Nea Madytos Health Center, Thessaloniki, GRC; 2 Primary care, Nea Madytos Health Center, Thessaloniki, GRC; 3 Primary care, 6th TOMY (Topical Organised Medical Units), Thessaloniki, GRC; 4 Gastroenterology and Hepatology, Biokliniki Hospital, Thessaloniki, GRC

**Keywords:** health services, healthcare services, health insurance, primary care, family medicine

## Abstract

The healthcare system in Greece has undergone significant changes over the past 10 years. While there have been some positive developments, such as improvements in primary care and public health, there are still many challenges that need to be addressed.

One of the major changes in the Greek healthcare system over the past decade has been the impact of the country's economic crisis. The government has had to implement a series of austerity measures, including significant cuts to healthcare funding. This has had a negative impact on the availability and quality of care, particularly for those on low incomes or living in rural areas.

## Editorial

A strong primary healthcare (PHC) system is critical to face the health challenges of our time, and Greece is no exception. The aging of the population, the global epidemiological shift toward chronic non-communicable diseases, the ongoing COVID-19 pandemic crisis, the exacerbation of health inequalities among countries and within countries, and the progress in technology, knowledge, and medical science, all require governments to act to defend the health of the global population [1].

In Greece, the healthcare system is a mixed national and social insurance system, funded by taxes and social insurance. Public PHC is mainly provided by the public sector through rural and urban health centers, as well as by the private sector, including private clinics in urban and semi-urban areas. Over the years, various governments have made efforts to reorganize and modernize the Greek healthcare system [2].

In 2017, a law was enacted to define the general principles of the National Health System (NHS), as defined in the Alma-Ata Declaration [3]. This law aimed to provide comprehensive health services, including health promotion, disease prevention, diagnosis, treatment, rehabilitation, and palliative care, in a qualitative and safe manner, ensuring equal access, universal coverage, and continuity of care, respecting the uniqueness, needs, and desires of each individual and the community as a whole. The law also established two levels of primary healthcare units (PHCUs). The first level includes PHCUs and various peripheral clinics that provide family and systematic health monitoring, home care, counseling, patient education, referral to hospitals if necessary, and the development of interventions and actions in the community. The second level is made up of health centers, urban or non-urban, which provide specialized services such as ambulatory care, laboratory and imaging tests, emergency and urgent care services, maternal care, child care, specialized prevention, social medical services, and public health [3].

In 2019, another law was enacted, aiming at the reorganization of PHC for better serving the healthcare needs of the Greek population [3]. This law established a design and coordination committee for PHC within the Ministry of Health, with the responsibility of centrally planning the goals of PHCUs and monitoring the implementation of actions at a national level. There is also a governing committee for the local PHCUs, which is a decentralized administration that implements and evaluates actions at a local level.

The healthcare system in Greece has been a topic of political discussion and debate for many years. While the country has made significant strides in improving its healthcare system over the past few decades, there are still several areas that need improvement. One of the biggest challenges facing the Greek healthcare system is the lack of funding. The country has been hit hard by the economic crisis, and the government has had to cut back on its spending in many areas, including health care. This has resulted in a shortage of resources, including medical equipment, personnel, and facilities, which has had a negative impact on the quality of care [4]. Another issue facing the Greek healthcare system is access to care. Many Greeks, particularly those living in rural areas or on low incomes, struggle to access the care they need. This is due to a lack of healthcare facilities in these areas, as well as the cost of care [4]. While the Greek government does provide some subsidies to low-income families, these are often insufficient to cover the cost of medical treatment [2]. In addition to these challenges, the Greek healthcare system also faces issues with bureaucracy and inefficiency. Patients often have to wait months for specialist appointments, and there are often long waiting times for surgeries and other treatment procedures. This can be frustrating and stressful for patients and can have a negative impact on their health outcomes.

Despite these challenges, there are also some positive aspects of the Greek healthcare system. For example, the country has a strong tradition of family medicine, and many Greeks have access to a primary care physician who provides them with ongoing care and support. Additionally, the country has made significant progress in reducing rates of smoking and alcohol consumption, which has helped to improve overall health outcomes [3].

To improve the Greek healthcare system, there are several steps that need to be done. First, the government needs to invest more in health care, including increasing funding for medical equipment, personnel, and facilities. This will help to improve the quality of care and reduce waiting times for patients. Secondly, the government needs to focus on improving access to care, particularly in rural areas and for low-income families. This could involve building new healthcare facilities in these areas, as well as increasing subsidies for those who cannot afford medical treatment. Finally, the Greek healthcare system needs to become more efficient and streamlined. This could involve reducing bureaucracy, improving communication between different parts of the healthcare system, and introducing new technologies to improve patient outcomes.

Health coverage has three dimensions referred to as the cube of coverage. One dimension is the population covered, while the other two are the services covered and the costs covered [2]. In Greece, it does not cover 100% of the costs (for example, prescription drugs have a 25% co-payment), and while it covers most of the services, there are significant delays and deficiencies, particularly in dental care and diagnostic tools (many of which are provided by the private sector with co-payment). Health coverage is a critical aspect of any healthcare system, and the three dimensions of coverage (population, services, and costs) are all essential factors that determine the quality of care that people receive. In Greece, the coverage of services is generally good, although there are significant delays and deficiencies in certain areas, particularly in dental care and diagnostics, as mentioned above. On the other hand, the costs of care are not fully covered, with patients having to pay a portion of the costs, which can be a barrier to accessing care for some [2]. Overall, while Greece's healthcare system has some strengths, there is still room for improvement to ensure that all citizens have access to affordable and high-quality healthcare services and there are also many opportunities for improvement. By investing in health care, improving access to care, and increasing efficiency, the Greek government can help to ensure that all Greeks have access to the high-quality, affordable health care they deserve.

Despite the country's universal healthcare coverage, there are still significant disparities in access to care, particularly for vulnerable populations such as low-income families and those living in rural areas with long waiting times for specialist appointments and surgeries [3]. This can be frustrating for patients and can lead to delays in diagnosis and treatment. Greece has a shortage of hospital beds compared to other European countries [3]. This can lead to overcrowding and delays in care, particularly during periods of high demand. For that reason, there have been efforts to strengthen primary care in Greece, with an emphasis on providing comprehensive and coordinated care to patients. Additionally, there has been a focus on the use of electronic health records (EHRs) in primary care settings in Greece, which has helped to improve the quality and efficiency of care. EHRs allow healthcare professionals to easily access and share patient information, which can lead to better coordination of care and improved patient outcomes [3].

Overall, the Greek healthcare system has many advantages and there are also areas where improvements are needed [1]. By addressing funding challenges, improving access to care, reducing bureaucracy and inefficiency, and investing in hospital infrastructure, the Greek government can help to ensure that all citizens have access to high-quality, affordable healthcare services. Over the last decade, primary care settings in Greece have undergone significant changes, with both positive and negative impacts on the healthcare system [2]. Another challenge in conclusion facing primary care in Greece is the impact of the economic crisis on the healthcare system. The crisis has led to significant cuts in healthcare spending, which has had a negative impact on the quality and availability of care. This has made it even more difficult for patients to access the care they need, particularly those who are most vulnerable.

In conclusion, while there are many positive aspects of the system, there are also areas where improvements are needed. The reorganization and modernization of the Greek PHC system are essential for improving the health outcomes of the Greek population, reducing health inequalities, and promoting universal coverage and continuity of care. It is important to ensure that the PHC system has the necessary organizational characteristics and service delivery, including governance, economic conditions, workforce development, accessibility, continuity, coordination, and comprehensiveness. This will require sustained political will, strong leadership, and significant investments in infrastructure, technology, and human resources. General practitioners need to be more educated in telemedicine and other edging technologies. Also, the physicians addressing medicine in rural and remote areas should receive economic benefits for their immense effort. The current and future governments must prioritize the building of a strong PHC system to meet the health needs of the Greek population. To address these challenges, there is a need for continued investment in primary care in Greece, with a focus on improving access to care, strengthening the healthcare workforce, and minimizing the impact of the economic crisis on the healthcare system.
